# Talent management of library and information science professionals: A review of research and future directions

**DOI:** 10.12688/f1000research.151301.2

**Published:** 2024-08-13

**Authors:** Bijayalaxmi Rautaray, Dillip K Swain, Chandrakant Swain

**Affiliations:** 1 Department of Library and Information Science, Kalinga Institute of Industrial Technology, Bhubaneswar, Odisha, India; 2 Library, Indian Institute of Management Raipur, Raipur, Chhattisgarh, India

**Keywords:** Talent management, career growth, organizational resilience, retention policy, employment strategy, talent pool

## Abstract

**Background:**

This study aims to review the extant literature on talent management with the objective of influencing library and information management by addressing the key facets of talent management, such as talent management strategies, importance of career development, evaluation of talented employees, and organizational resilience.

**Methodology:**

Literature on the development of talent and career management was retrieved from various scholarly papers indexed in Scopus and Web of Science to have a meticulous literature review serving as the platform of the present study. In light of the authors’ observations, two models were developed. The literature provides precise information that talent management plays a decisive role in promoting organizational excellence invariably in all kinds of organizations in general and libraries in particular.

**Results:**

This study provides constructive recommendations for the implementation of effective talent management and retention policies for library and information professionals. Moreover, this study adds immense value to the corpus of existing literature to set a platform for the augmentation of library management in futuristic vision.

**Conclusion:**

This study provides constructive recommendations to policy makers and library administrators to foster talented employees for excelling library and information services for the next several decades.

## Introduction

Talent management is a key area of research that explores qualitative human resources for the potential growth of an organization. Following conventional practices, academic libraries usually take a longer period to select and recruit qualified and competent library professionals, and often fail to hire and retain talented candidates ( Raschke, 2003; Warraich et al., 2019; Collings & Mellahi, 2009; Collings, 2014). Therefore, the importance of talent management for library and information science (LIS) professionals is very much essential for promoting academic excellence of institutions transcending the conventional practices. In the absence of a proper recruitment policy, many libraries randomly select and appoint average or below-average professionals for whom the respective institutions fail to obtain the desired results. Hence, extant literature on talent management needs to be meticulously comprehended as it is inevitable to develop a certain policy of talent management for library and information professionals looking at the holistic growth and development of the organization in line with its vision and mission.

### Aims and objectives

• To express the need for talent management in Library and Information Science (LIS) professional practice;
• To look at specific areas of library and information practices that can be considerably augmented by recruiting talented LIS professionals;
• To predict the possible output of talent management;
• To develop a schema for talent management strategy that can be followed suit by organizations as the essential attributes of the recruitment policy; and
• To put spotlight on healthy retention policy.

## Methods

Literature pertaining to the development of talent and career management was retrieved from various scholarly papers indexed in Scopus and Web of Science to have a meticulous literature review serving as the platform of the present study. Essential knowledge about global trends in the strategies for career management and talent development of employees was conceived. Scholarly documents were searched differently through the use of keywords like, “talent AND management”, “talent AND library professionals”. Though there were more than 1000 of articles traced from all branches of knowledge, we focussed only on journal articles ranging from 2010 which were partially related to the field of library and information science. In total, we reviewed 61 articles that included a few studies published before 2010. In light of the authors’ keen observation, two models were developed, and the authors discussed the key facets of career management of library and information science (LIS) professionals that are essential for augmenting the value of a given organization to justify returns on investment.

### Literature review

Existing literature primarily addresses two key aspects of talent management: inclusive and exclusive. The inclusive approach to talent management considers all employees ( Malik and Singh 2022; Cappelli and Keller, 2014; Gallardo-Gallardo et al., 2013; Iles et al., 2010), while the exclusive approach is designed to groom and foster only a few talented employees for which organizations are investing more in their talented employees ( Holck and Stjerne, 2019).

In total, there have been several studies on talent management, and the extant literature provides the conventional pattern of managing talent, identifying competent employees, and upgrading their positions and pay-perks ( DeVaro, 2006; Brewer, 2004; Nawe, 1992; Lewis and Heckman, 2006; Tarique and Schuler, 2010). Most studies have focused on explaining promotion criteria and how employees were engaged in competition among each other so that they could be promoted to the next higher level ( DeVaro, 2006). Claussen et al. (2014) stressed the key facets of talent management that constitute managerial skills and competencies ( Breaugh, 2011; Waldman, 2013).

Briscoe et al. (2006) posited that talent management should focus on the combination of boundary-less and protean career orientations as essential features of career profiles. Charbonneau and Freeman (2016) pointed out that the level of talent varies from person to person, which could influence the process of recruitment as far as academic libraries are concerned. They recommended that the academic libraries need to streamline the process of recruitment and hiring with a focus on retaining talented personnel for the greater interest of the organization. Yang (2022) examined key aspects of talent management in regard to innovation and sustainability. Vatousios and Happonen (2022) revealed the modus operandi of human resource management of organizations in managing talent based on qualitative analysis. However, the identification of such skills is crucial from the talent management perspective ( Collings & Mellahi, 2009).

Nawe (1992) discussed major issues related to the incorrect administration and mismanagement of talented professionals. Moreover, he explained the main reasons, including the wrong and haphazard recruitment policy. Annis (2003) pointed out staff concerns about career development in the promotional dimension. Chapman (2009) and Brewer (2004) advocated that there is a need for orientation programs among the employees of a given library to impart sufficient skills to retain their jobs by exhibiting talent in regard to transferring the tacit knowledge of senior professionals to freshly recruited ones. Li et al. (2018) provided constructive recommendations to resolve issues and problems related to talent management.

Kapardi, Supriyanka, and Balaji (2023) pointed out that for any institution, organization, or firm, employees are real assets. The study advocated that scopes and opportunities for career development must be explored, and employees must be given the right remuneration for their jobs, awards, and recognition in cases. Similarly, Ramirez-Lozano et al. (2023), Kongrode et al. (2023), and Bouteraa and Bouaziz (2023) argue that talented employees could ensure higher output if effective talent management designs and strategies were adopted. Although several studies have offered insights into the process, standards, and mechanisms of competition in promotion decisions, no comprehensive case study on grooming and promotion of talented LIS professionals towards the holistic growth, vision, and mission of the organization has been dealt with. Therefore, there is a research gap in this direction.

### Outcome of talent management

**Figure 1.**
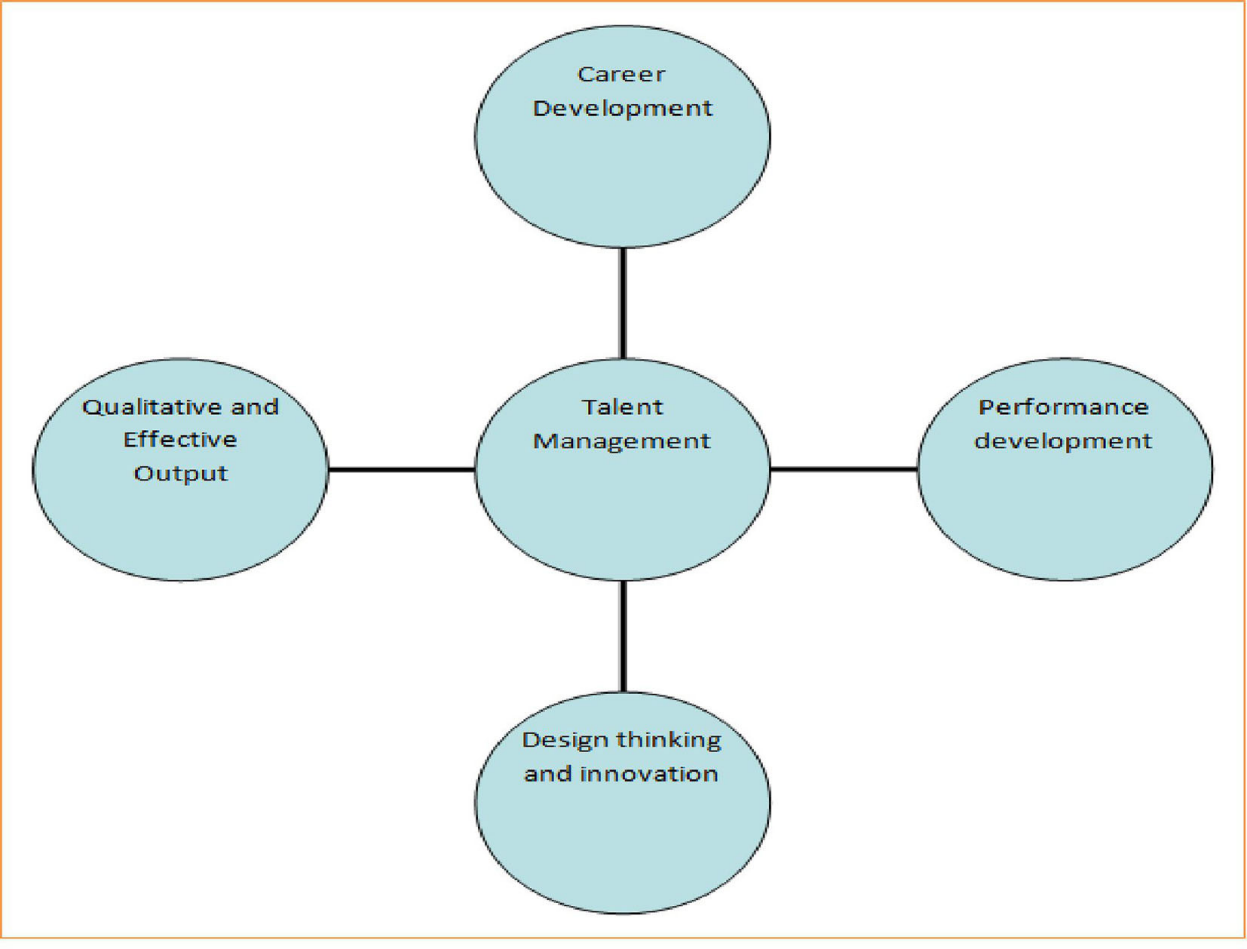
Outcome of talent management.

A good organization or firm believes in returns on investment. If talent management is strictly followed by all libraries of the world, it can ensure that the following key outcomes are depicted in Figure 1:

Figure 1 shows that talent management can promote the career development of LIS professionals by sponsoring them to participate in workshops and national and international conferences. Second, it can help to arrange special hands-on training programs based on the latest information communication technologies to ensure performance development of identified talented LIS professionals. Third, a few talented LIS professionals could be motivated to embrace ideas of creativity, design thinking, and innovation and put them into practice. Fourth, talent management should focus on qualitative output by setting proper strategies and benchmarks based on periodic evaluation of returns on investments.

### Talent management strategies

According to Yildiz and Esmer (2023), talent management begins with thorough planning with strategies that include talent identification, talent acquisition, and talent retention. Hence, human resource personnel administering libraries must formulate key strategies for effective talent management. With reference to the knowledge gained from the extant literature, the following key aspects must be considered while undertaking strategic talent management measures.

• Identification of talents
• Salary
• Duties and responsibilities
• Assigning responsibilities
• Periodic review of performance
• Incentives and annual increments
• Promotion based on performance

**Figure 2.**
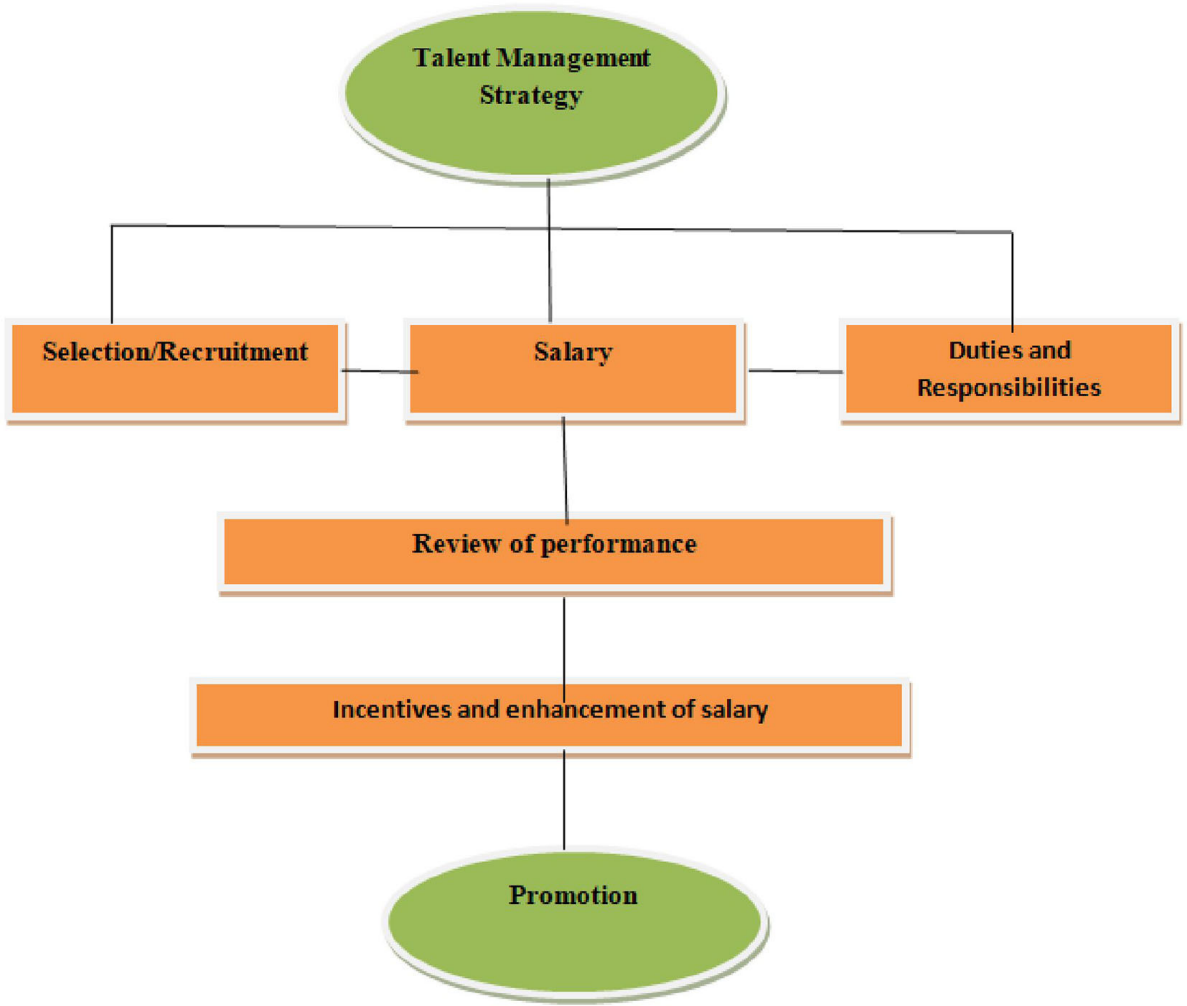
Talent management strategies.

Figure 2 shows that talent management strategy constitutes three major aspects: selection and recruitment, salary commensurate with qualifications and experiences of the employee, and allocation of duties and responsibilities. The next step is to review the performance of employees in line with the objectives and goals of the library, followed by incentives and enhancement of the salaries of the deserving candidates. The endpoint of the talent management strategy should be the promotion of talented employees.

### Evaluate candidates for organisational fit

It is crucial to select and recruit talented employees to meet the requirements of a specific organization, firm, library, or institution. Employees must showcase their potential in all possible dimensions ( Lyons et al., 2015; Kuron et al., 2016; Samanta et al., 2023). Therefore, it is essential to select and recruit employees who fit in their organization’s culture to successfully translate the vision and mission of the organization effectively and efficiently by justifying their performance to the best of their capabilities. Therefore, people at the top of human resource management must make the right judgment to select the fittest candidates for a specific unit of the organization.

### Focus on talent

It is essential to focus on talent, as talented employees are the true assets of a library and the organization. As pointed out by Garrow and Hirsh (2008), the focus of talent management essentially relates to organizational needs and requirements in different areas of the workforce, units, and sections that need to be properly addressed ( Garrow and Hirsh, 2008). Unless the focus on talent is correctly put, there is every chance of mismanagement and less return with poor output, as talent management involves systematic planning, performance monitoring, management, and periodic performance appraisal ( Meyers et al., 2020; Aljbour, French, and Ali, 2022).

Garrow and Hirsh (2008) remarked that talent management can be viewed with several kinds of focus within the organization, as both skill and career development are the essential components that set certain guidelines for the process of recruitment. Hence, the focus on talent should be judicious, perceptive, and value laden.

### Establishing new talent pool

In the words of Jyoti and Rani (2014), people who have the capabilities and high potential to enable firms to ensure effective output, outperform competitors, and achieve success in all possible dimensions through innovative ways of doing things are called talents. Therefore, it is very much crucial on the part of talent management team to establish a new talent pool banking on the young and energetic people who can serve the institution sincerely, complicatedly and relentlessly. Hence, planning and the policy of retention of talent should be judicious and talented employees need to be mentored.

Yarnall (2011) observes that the trends in talent management primarily focus on selecting and developing discrete pools of talent from within the selected units of an organization, and attention to the whole organization is not properly given. Therefore, it is essential to develop a talent pool by leaving no stones unturned.

Through a talent pool strategy, institutions can successfully meet their current and future competency needs by focusing on employees’ career needs and growth ( Sharma & Bhatnagar, 2009). There must be some strategies regarding the retention challenges of key talent while establishing a new talent pool ( Collings & Mellahi, 2009), especially the qualitative management and administration of libraries.

When establishing new talent pools, it is essential to improve succession-planning processes by creating a certain structure and pipeline for future roles ( Byham et al., 2002). Other dimensions include focusing on training and development and fostering human resources by considerably reducing turnover and retention of top talent. Thus, it is expected that personnel included in the talent pool should exhibit and prove their credentials and show that they are different from employees outside the pool ( Pepe, 2007; Yarnall, 2011; Seopa et al., 2015). Moreover, professional engagement as a form of professional development and the awareness of talented employees on the concept of returns on investment must be ensured. Keeping in the vision and mission of libraries of the future, it is crucial to identify talented employees and groom the new talent pool.

### Organisational resilience

Talent management must focus on organizational resilience. Lengnick-Hall et al. (2011) argued that talented employees must face all adverse situations with determination and robustness. Organizations need to develop a team with resilience capacity that enables them to be proactive to adverse effects and unexpected events, and to capitalize on events that could potentially threaten an organization’s survival ( Vogus and Sutcliffe, 2007; Lengnick-Hall et al., 2011).

Resilience is a dynamic process wherein individuals show courage, mental strength, and ability to “bounce back” from adversity or personal setbacks and even to grow and strengthen as a result of this adjustment ( Luthar & Cicchetti, 2000; Luthans et al., 2006; Williams et al., 2017). Resilience focuses on personality traits, personal characteristics, and environmental factors found in people to overcome risk factors ( Richardson, 2002; Duit, 2016). It should be noted that resilient people are often found with a positive outlook, self-esteem, good problem-solving skills, innovation with critical thinking skills, self-determination with perseverance, and above all amicable by nature ( Garmezy, 1991; Dyer and McGuiness, 1996; Cheese, 2016). Therefore, it is crucial to examine this aspect while selecting, fostering, and managing talented employees. The theoretical implications of organizational resilience may be incorporated into the policy of recruitment, fostering, and management of talents of library and information professionals.

### Employment strategy

Employment strategy is the essence of talent management. Advertisements for some posts should be meticulously performed. It should exclude or discourage applicants who are qualified from different institutions or have acquired experience from small organizations. As pointed out by Dalton et al. (2000), some government sectors exclusively advertise internal candidates by excluding talented applicants of LIS staff from other institutions. Similarly, Chatterjee et al. (2023) attribute the greater success of multinational enterprises that attract people from diverse backgrounds and different locations around the globe to ensure the selection of highly talented candidates for different jobs. Hence, there must be an efficient strategy to recruit talented candidates for different positions, either in the LIS or in any different field.

### Retention policy

Retention is one of the major issues found in all organizations, for which they fail to meet the desired level of output with a very poor turnover rate. Therefore, libraries need to strengthen their policy of retention and redesign retention programs to achieve excellent productivity and impressive turnover ( Huang Chuang, 2006; Deery & Jago, 2015). As recruiting employees and expecting high returns through wonderful performance is always doubtful, the retention of an experienced and motivated employee is crucial for the success of an organization. Hence, policymakers and managers of libraries and information centers must design effective policies for employee retention to ensure the following three key benefits:

• **Reduced cost**-It is found: Inexperienced employees often cause a reduction in the quality of services and loss of revenue. Therefore, it is essential to adopt an effective retention strategy to save time and money for the organization.
• **Competitive advantage**-Retention policy helps the organization achieve a competitive advantage over other organizations as they excessively bank on the excellent performance of experienced employees, and
• **Increased productivity-**If: If a proper retention policy is followed, employees get due motivation and start performing better.

### Practical implications

Library and Information Science is a field that has recently been regarded as one of the promising disciplines, as it leads from the front with respect to knowledge dissemination, storage, processing, information management, and control in a way that relates to the domain of all subjects and the universe of knowledge. Moreover, by mapping the productivity of individual researchers and institutions, bibliometric studies across disciplines have rightly sharpened the acumen of researchers to pursue their research in the right direction. Additionally, the LIS profession has a huge scope for studying, from the diploma level to doctoral and post-doctoral programs. A person with a library science degree can grow by leaps and bounds, beginning from library trainees to senior-level managerial positions. There are many opportunities for LIS professionals to obtain jobs in any sector. Therefore, talented library professionals need to be properly managed, groomed, and fostered in light of talent management methods and practices followed by some established and reputed organizations.

Continuing professional development is crucial for professionals’ successful career planning and prospects **.** Continuous hands-on training on the latest information communication technologies is essential to equip them to handle advanced learners and researchers, and to improve and develop various kinds of professional skills and technological competencies ( Dalton et al., 2000; Nankivell & Shoolbred, 1997). They need to be grounded in acquiring adequate technical, managerial, communication, presentation, leadership, and time management skills. In light of knowledge gained from the literature review, talented employees must be sponsored to attend various national seminars, international conferences, and workshops to enrich their acquired knowledge and experiences.

## Discussion

Current talent management research is rapidly moving towards exploring novel ways of managing talented people to ensure greater output to firms and organizations. In the aforementioned direction, scholars are reviewing the extant literature and spending a lot of time with practitioners to observe cases and best practices. The future of talent management promises to be brighter and more effective by charting a consistent pathway. Although earlier studies provide some managerial and practical implications for managers and talent management professionals, managing high-performance employees remains a challenge ( Scullion and Collings, 2011; Gallardo-Gallardo, 2019). To gain a competitive advantage, many firms and organizations have reduced their spending on employees in response to economic recession, but this adversely affects the reputation of firms and organizations ( Martin & Schmidt, 2010; Ehrnrooth et al., 2018; Sukla, 2023). Therefore, the need of the hour is to ensure healthy and effective talent management as per modern trends and future needs of libraries and organizations as time demands. It’s a million-dollar question how or why libraries should be competitive? Libraries must stay on top as there has been a great revolution in information communication technologies and vast changes in media consumption. Therefore, libraries being the service industry must be competitive and library professionals must strive to give their patrons what they need precisely and comprehensively in an efficient manner.

It was found that the active participation and involvement of employees in various training programs conducted outside their organizations fetches a moral boost that could enhance their ability to absorb, digest, and use new knowledge effectively. Therefore, talent management broadens the outlook and horizons of collaborators, and provides a solid basis for the acquisition, processing, and dissemination of new knowledge ( Latukha & Veselova, 2019). In summary, talent managers need to pay attention to the visions and goals of firms, organizations, libraries, or institutions of any kind.

### Scope for future studies

Much has been contemplated in theoretical parlance; now, the focus needs to shift to practical implications and case studies through rigorous research. The conceptual framework, strategy, and functions of talent management of library and information science professionals can be strengthened through empirical evidence based on case studies on talent management in specific organizations. This burning topic provides scope for plenty of research, including the following:

1. Research on talent management in reputed libraries of a specific region or country may be carried out with respect to employment strategy, organizational resilience, and retention policy, including other key facets of talent management.
2. A comparative study of talent management of libraries versus corporate/firms may be undertaken to elicit this gap.
3. A cross-cultural study on talent management can be conducted.

## Conclusion

Extant literature provides precise information that talent management plays a decisive role in invariably promoting organizational excellence in all kinds of organizations in general and libraries in particular. If the high wastage level of qualified and experienced LIS professionals is to be addressed, it is crucial to provide justice to talented candidates through an effective policy of recruitment with proper attention to career development.

 As discussed, talent succession planning, identifying new talent pools, and forming an effective strategy for recruitment and talent development are very important to increase productivity and enhance the image and reputation of organizations. The implementation of talent management practices is expected to enhance organizational resilience.

This study has a few limitations regarding the inclusion and exclusion criteria, as it is purely based on a review of the selected extant literature. Future studies could address this limitation by including more databases and resources during the search process.

### Ethics and consent

Ethical approval and consent were not required.
