# Supplementary material for: Talent management of library and information science professionals: A review of research and future directions
Source: F1000Res. 2024 Aug 13;13:652. Originally published 2024 Jun 18. [Version 2] doi: 10.12688/f1000research.151301.2 (PMC11344875; doi:10.12688/f1000research.151301.2)
Supplement: Peer review history

# Reviewer report: Reviewer response for version 2

**DOI:** 10.5256/f1000research.170162.r313810

**Published:** 2024-08-24

**Authors:** Derek Law

**Affiliations:** 1 University of Strathclyde, Glasgow, Scotland, UK

I am now happy to support the updated version of the article for indexing without reservations. The authors have clearly understood and fully addressed the comments made in my original review. I believe they have successfully revised the paper to take the comments into account both in terms of the specific comments on the use of terms such as "competitiveness" and also on the general style and use of a somewhat simpler vocabulary.

 I than have no additional comments and no reservations

Is the topic of the opinion article discussed accurately in the context of the current literature?

Yes

Are arguments sufficiently supported by evidence from the published literature?

Partly

Are all factual statements correct and adequately supported by citations?

Yes

Are the conclusions drawn balanced and justified on the basis of the presented arguments?

Partly

Reviewer Expertise:

Library and Information Science; Digital Humanities

I confirm that I have read this submission and believe that I have an appropriate level of expertise to confirm that it is of an acceptable scientific standard.

# Reviewer report: Reviewer response for version 2

**DOI:** 10.5256/f1000research.170162.r313809

**Published:** 2024-08-16

**Authors:** Razlina Razali

**Affiliations:** 1 Academy of Language Studies, Universiti Teknologi MARA Perlis Branch, Arau, Perlis, Malaysia

Thank you, authors for revising the manuscript. I have no further comments.

Is the topic of the opinion article discussed accurately in the context of the current literature?

Yes

Are arguments sufficiently supported by evidence from the published literature?

Partly

Are all factual statements correct and adequately supported by citations?

Partly

Are the conclusions drawn balanced and justified on the basis of the presented arguments?

Yes

Reviewer Expertise:

talent management, bibliometric analysis

I confirm that I have read this submission and believe that I have an appropriate level of expertise to confirm that it is of an acceptable scientific standard.

# Reviewer report: Reviewer response for version 1

**DOI:** 10.5256/f1000research.165938.r308041

**Published:** 2024-08-06

**Authors:** Razlina Razali

**Affiliations:** 1 Academy of Language Studies, Universiti Teknologi MARA Perlis Branch, Arau, Perlis, Malaysia

The topic of the study is interesting and has the potential to expand the body of knowledge on talent management (TM). The references used in the article also include those published in recent years. The following are comments for the authors to improve the quality of the article:

 1.   Explaining the importance of TM for library and information science (LIS) professionals in the introduction by citing related literature

 2. Expand the methods section by explaining how many papers were selected for the review and the selection criteria used such as the year, keywords, the types of articles etc.

 3. What are the recent studies on LIS professionals and what are their major findings? Why is there a need for a review study to be conducted on this topic?

 4. In the discussion section, authors need to reiterate the importance of TM for LIS professionals by relating it to the current trends in the field of study and practices. Please include current literature to discuss. 

 5. Please cite this statement correctly: Lengnick-Hall et al. (2011) emphasized that talented employees must have the “ability to rebound from unexpected, stressful, and adverse situations to bounce back in style with determination and robustness.

Is the topic of the opinion article discussed accurately in the context of the current literature?

Yes

Are arguments sufficiently supported by evidence from the published literature?

Partly

Are all factual statements correct and adequately supported by citations?

Partly

Are the conclusions drawn balanced and justified on the basis of the presented arguments?

Yes

Reviewer Expertise:

talent management, bibliometric analysis

I confirm that I have read this submission and believe that I have an appropriate level of expertise to confirm that it is of an acceptable scientific standard, however I have significant reservations, as outlined above.

# Response

**Published:** 2024-08-07

**Authors:** Bijayalaxmi Rautaray

**Affiliations:** Dept of Lib & Inf. Sc, KIIT Deemed to be University, Bhubaneswar, Odisha, India

**Esteemed Reviewer,**

 Thank you so much for the valuable remarks! We have tried our best to comply with the comments/suggestions:

**Reviewer 2 :** **Razlina Razali** **, Universiti Teknologi MARA Perlis Branch, Arau, Malaysia**

**Approved With Reservations**

**Sl No**

**Reviewers comments/suggestions**

**Compliance**

 1

 Explaining the importance of TM for library and information science (LIS) professionals in the introduction by citing related literature

**We appreciate the remarks of the esteemed reviewer. A glimpse of the importance of TM for library and information science is provided.**

 2

 Expand the methods section by explaining how many papers were selected for the review and the selection criteria used such as the year, keywords, the types of articles etc.

**Scholarly documents were searched differently using keywords like, “talent AND management”, “talent AND library professionals”. Though there were more than 1000 of articles traced from all branches of knowledge, we focused only on journal articles ranging from 2010 which were partially related to the field of library and information science. In total we reviewed 61 articles that included a few studies published before 2010.**

 3

 What are the recent studies on LIS professionals and what are their major findings? Why is there a need for a review study to be conducted on this topic?

**The findings of the recent studies on talent management of LIS professionals have been precisely discussed in the literature review. Owing to the growing importance of managing talents, pertinent related literatures have been reviewed**

 4

 In the discussion section, authors need to reiterate the importance of TM for LIS professionals by relating it to the current trends in the field of study and practices. Please include current literature to discuss.

**The esteemed reviewer’s remark is appreciated. However, we have put spotlight on a selected few recent studies like, Ehrnrooth et al., 2018, Latukha & Veselova, 2019: Gallardo-Gallardo, 2019: Sukla, 2023. Hence, we have thoughtfully made the discussion part quite precise to make the text impactful.**

 5

 Please cite this statement correctly: al. (2011) Lengnick-Hall et emphasized that talented employees must have the “ability to rebound from unexpected, stressful, and adverse situations to bounce back in style with determination and robustness.

**Done**

 Best regards

# Reviewer report: Reviewer response for version 1

**DOI:** 10.5256/f1000research.165938.r294732

**Published:** 2024-07-16

**Authors:** Derek Law

**Affiliations:** 1 University of Strathclyde, Glasgow, Scotland, UK

This is an interesting if basic literature review of papers on talent management and career development. It is comprehensive in scope and analyses the literature fully then goes on to develop two models for managing staff and their career progression. It also makes it clear that good organisational talent management plays a decisive role in promoting organizational excellence.

 Major issues. Although the literature review is well done and well discussed, the language used in the paper is very overblown, very otiose and can be very repetitive, e.g.  "extant" and "promotional development" and other words or phrases are used very many times. Some phrases seem to be used wrongly, e.g. "rooms should be created" or "moral boost". In this case "mentoring" is a much better word. There is a need for significant language editing of the text.

 A second issue is that although it is not a central to the argument, there is no explanation of how or why libraries should be competitive – a curious concept.

 Minor:  Almost nothing is said about professional engagement as a form of professional development. It would be useful to have a sentence or two on this.

 Secondly it would be interesting to explain how the concept of mentoring has developed into the new phrase of talent management.

 In sum it is a useful literature review which provides a good summary of current thinking, but really needs extensive editing and a little more work to explain the minor items mentioned above.

Is the topic of the opinion article discussed accurately in the context of the current literature?

Yes

Are arguments sufficiently supported by evidence from the published literature?

Partly

Are all factual statements correct and adequately supported by citations?

Yes

Are the conclusions drawn balanced and justified on the basis of the presented arguments?

Partly

Reviewer Expertise:

Library and Information Science; Digital Humanities

I confirm that I have read this submission and believe that I have an appropriate level of expertise to confirm that it is of an acceptable scientific standard, however I have significant reservations, as outlined above.

# Response

**Published:** 2024-08-07

**Authors:** Bijayalaxmi Rautaray

**Affiliations:** Dept of Lib & Inf. Sc, KIIT Deemed to be University, Bhubaneswar, Odisha, India

**Esteemed Reviewer,**

 Thank you so much for a meticulous review of our manuscript. Your insightful remark has augmented the value of this paper.

**Reviewer 1:** **Derek Law** **,** **University of Strathclyde, Glasgow, Scotland, UK** 

**Approved With Reservations**

**Sl No**

**Reviewer's comments/suggestions**

**Compliance**

 1

 Although the literature review is well done and well discussed, the language used in the paper is very overblown, very otiose and can be very repetitive, e.g.  "extant" and "promotional development" and other words or phrases are used very many times. Some phrases seem to be used wrongly, e.g. "rooms should be created" or "moral boost". In this case "mentoring" is a much better word.

 We sincerely appreciate the esteemed reviewer’s remarks. Thanks and regards

**We have made changes as advised.**

 2

 There is a need for significant language editing of the text.

 Thanks a lot.

**The manuscript has been checked by a language professor**

 3

 There is no explanation of how or why libraries should be competitive – a curious concept.

**Added at the end of 1 ^st^ Para of discussion**

 4

 Almost nothing is said about professional engagement as a form of professional development. It would be useful to have a sentence or two on this.

***Mentioned in the facet, “establishing a new talent pool”***

 5

 It would be interesting to explain how the concept of mentoring has developed into the new phrase of talent management.

**Done**

 Hope, we have tried our best 

 Best regards
